# No Evidence of HTLV-II Infection Among Immonoblot Indeterminate Samples Using Nested PCR in Mashhad, Northeast of Iran

**Published:** 2013-03

**Authors:** Houshang Rafatpanah, Farhad Fathimoghadam, Majid Shahabi, Iman Eftekharzadeh, Mohammadreza Hedayati-Moghaddam, Narges Valizadeh, Mohsen Tadayon, Seyyed Aliakbar Shamsian, Hamidreza Bidkhori, Raheleh Miri, Ali Bazarbachi

**Affiliations:** 1 Research Centre for HIV/AIDS, HTLV and Viral Hepatitis, Iranian Academic Centre for Education, Culture & Research (ACECR), Mashhad Branch, Mashhad, Iran; 2 Inﬂammation and Inﬂammatory Diseases Research Centre, School of Medicine, Mashhad University of Medical Sciences, Mashhad, Iran; 3 Research Centre for the Iranian Blood Transfusion Organization (IBTO), Tehran, Iran; 4 Faculty of Medicine, Islamic Azad University, Mashhad Branch; 5 Department of Internal Medicine, American University of Beirut, Beirut, 10 Lebanon

**Keywords:** HTLV-I, HTLV-II, Iran, Mashhad, PCR, Seroindeterminate, Western blot

## Abstract

***Objective(s):*** Although HTLV-I infection is endemic in different geographical parts of the world including Northeast of Iran, there have been no documents of HTLV-II infection in this region. It is reported that one possible reason for seroindeterminate state in HTLV western blot is HTLV-II virus. This study aimed to investigate the presence of HTLV-II among blood donors with seroindeterminate western blot results.

***Materials and Methods:*** Three ml whole blood obtained from 50 blood donors referring to Mashhad Blood Transfusion Organization who had reactive Elisa for HTLV-I and seroindeterminate HTLV western blot state. A conventional PCR was applied to detect HTLV-I provirus using specific primers while a nested PCR was designed with specific external and internal primers for the detection of HTLV-II.

***Results:*** The average age of participants, 39 males and 11 females, was 37.12± 14.36 years. The average OD of the Elisa assay was 1.767± 1.195. The most common indeterminate patterns were Rgp46-II alone (n=12, 27.3%), Rgp46-I alone (n=7, 15.9%), and Rgp46-I with GD21 (n=7, 15.9%).After introducing the DNA to the PCR tests, results revealed 10 (20%) HTLV-I PCR positive samples while no HTLV-II positive sample was detected by nested PCR. There were no significant age, blood group, Optical Density of the Elisa assay, and western blot indeterminate pattern differences between HTLV-I PCR positive and negative samples.

***Conclusion*** ***:*** No HTLV-II positive sample was detected in this study which confirms the absence of HTLV-II infection in this region. However, high frequency of HTLV-I PCR positive samples among the seroindeterminate cases implies on the important role of molecular techniques for further confirmation of the infection.

## Introduction

Human T-Lymphotropic Virus (HTLV) types I and II were the first discovered human retroviruses. HTLV-I was first reported in 1980 while HTLV-II was first reported in 1982 (1-3). HTLV-I is believed to be an etiologic factor for Adult T-cell Leukemia (ATL) and HTLV-I Associated Myelopathy/ Tropical Spastic Paraparesis (HAM/TSP) (4-7). However HTLV-II has not been directly connected to any human disease although some recent studies indicate that HTLV-II infection may be associated with two distinct neurologic disorders with similar HAM/TSP symptoms and Tropical Ataxic Neuropathy (TAN) (8-10).

HTLV-I is endemic in a number of geographical regions including Japan, Africa, Caribbean islands, South America, and Northeast Iran while the epidemiology of HTLV-II is not well identified (11-15).

Within the last decade newly developed serologic assays made us capable to accurately differentiate between antibodies to HTLV-II and HTLV-I. These new techniques include synthetic Enzyme Immunoassays (EIA) (16, 17) and recombinant proteins in a modified Western blot (WB) (18-20).

Although such techniques greatly improve the identification and differentiation of HTLV-II from HTLV-I a frequent problem in HTLV-I/II diagnosis which is the high prevalence of indeterminate serological results by WB still leads to difficulties in the interpretation (21).

The current diagnosis of HTLV-I and HTLV-II infection is mainly based on Enzyme-linked immunosorbent assays (ELISA) followed by the confirmation of ELISA reactive samples with WB analysis (22). To distinguish HTLV-I from HTLV-II the incorporation of recombinant type-specific glycoprotein rgp46-I or rgp46-II on the WB assay would be employed (22, 23). However some individuals with positive ELISA results show incomplete antibody reactivity to HTLV-I or HTLV-II whom are considered to have indeterminate WB status (24-26). Some recent studies identified HTLV-II proviral DNA in WB indeterminate samples using nested Polymerase Chain Reaction (PCR) analysis (21, 27, 28).

Mashhad, located in Northeast of Iran, is an endemic region for HTLV-I infection. It has been recently reported that 2.12% of the general population and 0.77% of blood donors were infected with HTLV-I (15). However no data regarding the situation of HTLV-II infection in this geographical region have been reported yet. Screening of blood donors of Mashhad for HTLV infection which has been mandatory since 1996, is currently based on ELISA assay followed by confirmatory WB analysis (29).

To better understand the molecular epidemiology of HTLV infection in this endemic area better, it would be quite beneficial to determine the proportion of HTLV-I/II infection among HTLV-seroindeterminate individuals. The current study aimed to investigate the presence of HTLV-II and HTLV-I infections in Mashhad among WB indeterminate samples from blood donors by nested PCR.

## Material and Methods

In this descriptive cross-sectional study blood samples of 50 subjects with WB indeterminate result for HTLV collected from Khorasan Blood Transfusion Organization (BTO), who had donated blood during 2009 and 2010 were selected.

**Figure 1:**
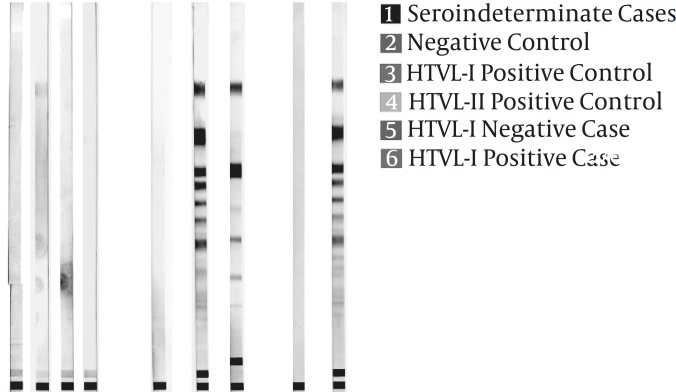
Different WB interpretations among the study samples

Serum samples of all blood donors in Khorasan BTO were routinely tested for HTLV-I/II antibodies using ELISA assay (MP HTLV-I/II ELISA 3.0, MP Diagnostics, Singapore) according to the manufacturer’s instructions and reactive samples were reevaluated in duplicate with the same kit. Repeatedly reactive samples were further evaluated for confirmation of the infection with a WB assay (MP HTLV 2.4 Western blot, MP Diagnostics, Singapore) following the manufacturer’s criteria. According to World Health Organization (WHO) criteria for HTLV seropositivity in WB assay, a sample would be regarded as positive when either gp46 or gp462/68 and one of p19, p24, or p53 were present. The HTLV European Research Network proposed the stringiest criteria for WB seropositivity that defines a WB assay as HTLV-I positive only if bands for the gag proteins p19 and p24 and the env proteins gd21 and MTA-1 were present while it scored as HTLV-II positive if p24, gd21, and K-55 bands were identified and was HTLV positive but nontyped if p24, p19, and gd21 were present (25). In the current study the criteria issued by MP diagnostics HTLV blot 2.4 was employed which is more similar to the HTLV European Research Network criteria. In brief the positive WB for HTLV-I is regarded as the presence of group-specific antigen (gag) proteins (p19 with or without p24) and two envelope (env) proteins (gd21 and rgp46-I) while positive HTLV-II has bands for the gag proteins (p19 with or without p24) and two env proteins (gd21 and rgp46-II). The presence of p24, p19, and gd21 was considered as nontyped HTLV positive. The test was defined as indeterminate if specific bands for HTLV that did not meet the criteria of positivity were present. Besides, those samples that did not show specific bands for HTLV were regarded as negative (Figure 1).

To perform the molecular analysis, 3 ml whole blood was collected in sterile Ethylenediaminetetraacetic acid (EDTA) tubes and genomic DNA was extracted from peripheral blood of WB indeterminate individuals using a commercial DNA extraction kit (QIAmp DNA blood minikit, Qiagen Gmbh, Hilden, Germany) according to the manufacturer’s instructions.

Extracted DNA stored at -20 until PCR analysis. In order to confirm HTLV-I infection, Long Terminal Repeat (LTR) and TAX genes were targeted and amplified using designed specific primers (Table 1).

**Table 1:** Specific primers designed for TAX and LTR regions

Name	Sequence(5-3)	Position	Amplicon (base pair)
Taxf	AGGGTTTGGACAGAGTCTT		
Taxr	AAGGACCTTGAGGGTCTTA	7335-7590	256 Bp
LTRf	CATAAGCTCAGACCTCCGGG		
LTRr	GGATGGCGGCCTCAGGTAGG	8107-8330	224 Bp

PCR condition for LTR and TAX genes were 10X buffer 2.5 µl, Mgcl_2_, 2 µl (final concentration of 2 mM), dNTPs 0.5 µl (final concentration 0.2 mM), 0.5µl of each primers (10mM) , 0.5 unit of Taq DNA polymerase enzyme (GENETBIO, Korea) and 50-100 ng DNA. The PCR machine (Astech, JAPAN) was programmed to 94˚C 5min, 35 cycles of 94˚C 45 sec, 60˚C 50 sec, 72˚C 50 sec and a final extension of 72˚C 7min.

Amplified products were introduced on 2% gel agarose, stained with ethidium bromide and observed under ultraviolet (UV) luminometer.

 To determine HTLV-II infection the regions of LTR and TAX genes were selected and amplified using designed specific primers (Table 2).

LTR fragment was amplified by a nested PCR. Briefly a first-round PCR reaction was carried out on 50-100 ng of extracted DNA in a 25 µl reaction mixture containing 0.5 U of Taq DNA polymerase (Genetbio, Korea) 2.5 µl 10x buffer, 2 mM MgCl_2_, 0.2 mM of each of the Deoxynucleoside triphosphates, and 10 pmol of each outer primer. The PCR cycling conditions consisted of 35 cycles at 95°C for 45 sec, 58°C for 60 sec, and 728C for 180 sec and a final extension of 72˚C for 7min.

**Table 2:** Inner and outer specific primers designed for TAX and LTR regions used in the nested PCR

	Name	Sequence(5’-3’)	Position	Genbank	Amplicon(Base pair)
First set	LTR 2F1 LTR 2 R1	CTAGCCTCCCAAGCCAGCCACC CCAGTGGTGGGTTGATAGCCC	13-878	M10060	Outer 866 bp
Second set	LTR2 F2 LTR2 R2	CGAGTCATCGACCCAAAAGGTC GGAGTTGGGGAAAGCCCGTGG	40-838		Inner 799 bp
First set	tax2 F1 tax2 R1	TGGATACCCCGTCTACGTGT GAGCTGACAACGCGTCCATCG	7248-7406		0uter 159bp
Second set	tax2 F2 tax2 R2	CTACGTGTTTGGCGATTGTGTAC TGACAACGCGTCCATCGAT	7260-7402		Inner 143bp

For the second round 3µl of the first PCR product was used. The PCR mixture was the same as first round PCR but with inner primer set. The PCR machine (Astech, JAPAN) was programmed to 94˚C for 5 min, 35 cycles of 94˚C for 30 sec, 60˚C for 30 sec, 72˚C for 60 sec and a final extension of 72˚C for 7min.

A 159 base pair fragment of TAX gene was amplified by the outer primer (Table 2). PCR machine was programmed to 94˚C for 5 min, 35 cycles of 94˚C for 30 sec, 58˚C for 30 sec, 72˚C for 60 sec and a final extension of 72˚C for 7 min. 1 µl of the first PCR product was used for amplification of a 143 base pair fragment using inner primer (Table 2). The PCR machine was programmed to 94˚C for 5min, 35 cycles of 94˚C for 30 sec, 55˚C for 30 sec, 72˚C for 60 sec and a final extension of 72˚C for 7 min.

All amplified products were run on 2% gel agarose, stained with ethidium bromide and observed under UV luminometer.

The current study is approved by the research deputyship of Iranian Academic Centre for Education, Culture, and Research (ACECR) Mashhad branch regarding ethical and methodological issues. A written consent was obtained from each individual after describing the aims of the study and assuring the participants about the results to be remained anonymous.

Demographic data as well as WB and PCR results were registered and analyzed using SPSS V.18.0. Chi-square and Fisher’s exact tests as well as T-test were employed to perform categorical and numerical comparisons respectively. Related tables were produced to discuss the results. *P*-values <0.05 were considered as statistically significant.

## Results

In this study a total 50 WB indeterminate samples were investigated for HTLV-I and HTLV-II proviral DNA using nested PCR. From these 50 participants 39 (78%) were male and 11 (22%) were female. The mean age of participants was 37.12±14.36 years. The most and the least common blood groups were O^+^ and AB^-^ with 35.4% (n=17) and 0% respectively (Table 3).

**Table 3:** Frequency of blood groups among WB indeterminate individuals

Blood group	Number	%
A^+^	9	18.8
B^+^	16	33.2
O^+^	17	35.4
AB^+^	2	4.2
A^-^	2	4.2
B^-^	1	2.1
O^-^	1	2.1
AB^-^	0	0

All samples had HTLV-I/II positive reactivity in ELISA. The mean value of Optical Density (OD) in ELISA for the samples was 1.767±1.195.

The most common bands in WB were gd21 (47.7%), rgp46-I (38.6%), rgp46-II (31.8%), gp21 (9.1%), p24 (6.8%), p53 (2.3%), and p26 (2.3%) respectively.

WB indeterminate patterns were identified for all samples. The most common WB indeterminate patterns were rgp46-II alone (27.3%), rgp46-I alone (15.9%), rgp46-I and gd21 (15.9%), gd21 alone (11.4%), rgp46-I, p24, and gd21 (4.5%), rgp46-II and gd21 (4.5%), p24 and gd21(2.3%), rgp46-I, gd21, and gp21 (2.3%), and p53 alone (2.3%) respectively (Table 4).

**Table 4:** The frequency of Different WB indeterminate patterns among samples (n=44)

WB indeterminate pattern	Number	%
Rgp46-I	7	15.9
Rgp46-II	12	27.3
Rgp46-I,P24,GD21	2	4.5
GP21	5	11.4
Rgp46-I, GD21	7	15.9
GD21	6	13.6
Rgp46-II, GD21	2	4.5
P24, GD21	1	2.3
Rgp46-I, GD21, GP21	1	2.3
P53	1	2.3

Nested PCR revealed no HTLV-II proviral DNA in samples. However 10 samples (20%) were positive for HTLV-I. From the 10 HTLV-I positive individuals 8 were male (80%) and 2 were female. Also statistical analysis showed no significant difference between HTLV-I PCR positive and negative individuals regarding age, blood group, ELISA OD, and WB indeterminate pattern (*P*>0.05).

## Discussion

The use of PCR enabled scientists to directly detect proviral DNA sequences of HTLV-I and HTLV-II from HTLV infected patients (30, 31). However wide application of PCR was time consuming and expensive which is not suitable for the analysis of large numbers of blood samples (16, 32). Therefore although PCR remained the standard diagnosis of HTLV infection, it is not used as a routine diagnostic tool. Therefore WB assay is used to confirm HTLV-I/II infection in seropositive ELISA samples using recombinant proteins specific for HTLV-I/II env glycoproteins incorporated into WB strips. Such recombinant proteins increase the sensitivity of the assay and enable it to differentiate between HTLV-I and HTLV-II infections. The overall sensitivity and specificity of HTLV WB are reported as 97.1% and 97.5% respectively (33).

By the wide utilization of WB assay for HTLV confirmation WB indeterminate results became a global problem for blood banks specially in endemic areas (29).

Several possible explanations have been proposed for the occurrence of HTLV WB indeterminate states. The most accepted explanations include cross reacting to other known retroviruses like Simian T-Cell Leukemia Virus (STLV) or HTLV-I related endogenous sequence (HRES-I) or a novel virus, antibody responses to a malaria parasite with epitope homology to HTLV-II like Plasmodium Falciparum, a defective HTLV-I or HTLV-II, and low copy numbers of prototype HTLV-I in the affected patient having indeterminate state (33).

To better understand the status of HTLV-I and HTLV-II infections in WB indeterminate samples we investigate 50 indeterminate samples from Mashhad blood donors.

The current study results demonstrate a high rate of HTLV-I infection among WB indeterminate samples (20%) using PCR technique that may be due to low copy number of prototype HTLV-I. Similar results reported by previous studies indicated the ability to amplify HTLV-I tax region from PBMCs of some HTLV WB indeterminate individuals (26, 34). Moreover the current study results showed that the previous hypothesis that HTLV WB indeterminate samples could be considered as negative is wrong. Presence of 20% HTLV-I positivity among indeterminate WB samples displays a relatively high rate and emphasizes the importance of molecular investigations to detect HTLV as well as the importance of determining the exact status of WB indeterminate individuals to better control the infection. Similar results with various frequencies were reported by Costa and Segurado in Brazil, Mangano *et al* in Argentina, Berini *et al* in Argentina, and Santos *et al* in Brazil with 9.2%, 13.8%, 13.2%, and 22% frequencies respectively (21, 27, 28, 35).

Similar to other studies performed on WB indeterminate samples, the current study observed no significant difference regarding age, gender, and blood group of HTLV-I PCR positive and negative individuals (35, 36).

Nested PCR results showed no evidence of HTLV-II infection among indeterminate WB individuals in the present study which was predictable due to no previous reports of HTLV-II in this region (12, 15). Similar studies in other geographical regions especially South America revealed some cases of HTLV-II among WB indeterminate individuals (21, 27, 28, 37).

The most common indeterminate patterns in the current study were single bands of rgp46-II, rgp46-I, and gd21 which were similar to the findings of Mangano *et al* but in contrast to the findings of Rouet *et al* and Costa and Segurado (25, 27, 28).

## Conclusion

The results of the current study revealed a high rate of HTLV-I infection among WB indeterminate samples as well as no HTLV-II infection. Therefore it can be concluded that HTLV-II is not a matter of concern in this region while special attention to indeterminate WB individuals regarding HTLV-I infection is necessary. It is recommended to determine the status of HTLV-I infection in WB indeterminate blood donors in Northeast of Iran using molecular techniques such as PCR to control the infection.

## References

[1] Gallo RC (2005). The discovery of the first human retrovirus: HTLV-I and HTLV-II. Retrovirology.

[2] Mueller N (1991). The epidemiology of HTLV-I infection. Cancer Causes and Control.

[3] Poiesz BJ, Ruscetti FW, Gazdar AF, Bunn PA, Minna JD, Gallo RC (1980). Detection and isolation of type C retrovirus particles from fresh and cultured lymphocytes of a patient with cutaneous T-cell lymphoma. Proce Natl Acad Sci.

[4] Dahmoush L, Hijazi Y, Barnes E, Stetler-Stevenson M, Abati A (2002). Adult T-cell leukemia/lymphoma. Cancer Cytopathol.

[5] Izumo S, Umehara F, Osame M (2000). HTLV-I-associated myelopathy. Neuropathology.

[6] Matutes E (2007). Adult T-cell leukaemia/lymphoma. J Clin Pathol.

[7] Osarne M, Usuku K, Izumo S, Ijichi N, Amitani H, Igata A (1986). HTLV-I associated myelopathy, a new clinical entity. Lancet.

[8] Araujo A, Hall WW (2004). Human T-lymphotropic virus type II and neurological disease. Ann Neurol.

[9] Jacobson S, Lehky T, Nishimura M, Robinson S, McFarlin DE, Dhib-Jalbut S (2004). Isolation of HTLV-II from a patient with chronic, progressive neurological disease clinically indistinguishable from HTLV-I-associated myelopathy/tropical spastic paraparesis. Ann Neurol.

[10] Sheremata WA, Harrington Jr WJ, Bradshaw PA, Foung SKH, Raffanti SP, Berger JR (1993). Association of ‘(tropical) ataxic neuropathy’with HTLV-II. Virus Res.

[11] Hall WW, Ishak R, Zhu SW, Novoa P, Eiraku N, Takahashi H (1996). Human T lymphotropic virus type II (HTLV-II): epidemiology, molecular properties, and clinical features of infection J Acquir Immune Defic Syndr Hum Retrovirol.

[12] Hedayati-Moghaddam M, Fathimoghadam F, Mashhadi IE, Soghandi L, Bidkhori H (2011). Epidemiology of HTLV-I in Neyshabour, Northeast of Iran. Iran Red Crescent Med J.

[13] Lowis GW, Sheremata WA, Minagar A (2002). Epidemiologic Features of HTLV-II:: Serologic and Molecular Evidence. Ann Epidemiol.

[14] Proietti FA, Carneiro-Proietti ABF, Catalan-Soares BC, Murphy EL (2005). Global epidemiology of HTLV-I infection and associated diseases. Oncogene.

[15] Rafatpanah H, Hedayati-Moghaddam MR, Fathimoghadam F, Bidkhori HR, Shamsian SK, Ahmadi S (2011). High prevalence of HTLV-I infection in Mashhad, Northeast Iran: A population-based seroepidemiology survey. J Clin Virol.

[16] Lal R, Heneine W, Rudolph D, Present W, Hofhienz D, Hartley T (1991). Synthetic peptide-based immunoassays for distinguishing between human T-cell lymphotropic virus type I and type II infections in seropositive individuals. J Clin Microbiol.

[17] Rudolph DL, Lal R (1993). Discrimination of human T-lymphotropic virus type-I and type-II infections by synthetic peptides representing structural epitopes from the envelope glycoproteins. Clin Chem.

[18] Lillehoj E, Alexander S, Dubrule C, Wiktor S, Adams R, Tai CC (1990). Development and evaluation of a human T-cell leukemia virus type I serologic confirmatory assay incorporating a recombinant envelope polypeptide. J Clin Microbiol.

[19] Lipka J, Santiago P, Chan L, Reyes G, Samuel K, Blattner W (1991). Modified Western blot assay for confirmation and differentiation of human T cell lymphotropic virus types I and II. J Infect Dis.

[20] Roberts BD, Foung S, Lipka J, Kaplan J, Hadlock K, Reyes G (1993). Evaluation of an immunoblot assay for serological confirmation and differentiation of human T-cell lymphotropic virus types I and II. J Clin Microbiol.

[21] Berini CA, Eirin ME, Pando MA, Biglione MM (2007). Human T-cell lymphotropic virus types I and II (HTLV-I and -II) infection among seroindeterminate cases in Argentina. J Med Virol.

[22] Thorstensson R, Albert J, Andersson S (2002). Strategies for diagnosis of HTLV-I and -II. Transfusion.

[23] Yao K, Hisada M, Maloney E, Yamano Y, Hanchard B, Wilks R (2006). Human T lymphotropic virus types I and II western blot seroindeterminate status and its association with exposure to prototype HTLV-I. J Infect Dis.

[24] Garin B, Gosselin S, Gessain A (2005). HTLV-I/II infection in a high viral endemic area of Zaire, Central Africa: Comparative evaluation of serology, PCR, and significance of indeterminate Western blot pattern. J Med Virol.

[25] Rouet F, Meertens L, Courouble G, Herrmann-Storck C, Pabingui R, Chancerel B (2001). Serological, epidemiological, and molecular differences between human T-cell lymphotropic virus Type 1 (HTLV-I)-seropositive healthy carriers and persons with HTLV-I Gag indeterminate Western blot patterns from the Caribbean. J Clin Microbiol.

[26] Soldan SS, Graf MD, Waziri A, Flerlage AN, Robinson SM, Kawanishi T (1999). HTLV-I/II seroindeterminate Western blot reactivity in a cohort of patients with neurological disease. J Infect Dis.

[27] Costa JMP, Segurado AC (2009). Molecular evidence of human T-cell lymphotropic virus types 1 and 2 (HTLV-I and HTLV-II) infections in HTLV seroindeterminate individuals from São Paulo, Brazil. J Clin Virol.

[28] Mangano A, Remesar M, Del Pozo A, Sen L (2004). Human T lymphotropic virus types I and II proviral sequences in Argentinian blood donors with indeterminate Western blot patterns. J Med virol.

[29] Zanjani D, Shahabi M (2011). Molecular analysis of human T-cell lymphotropic virus type 1 and 2 (HTLV-I/2) seroindeterminate blood donors from Northeast of Iran; evidence of proviral tax, env, and gag sequences. Retrovirology.

[30] De BK, Srinivasan A (1989). Detection of human immunodeficiency virus (HIV) and human lymphotropic virus (HTLV) type I or II dual infections by polymerase chain reaction. Oncogene.

[31] Vrielink H, Zaaijer H, Cuypers H, Poel CL, Woerdeman M, Lelie P (2003). Evaluation of a New HTLV-I/II Polymerase Chain Reaction. Vox Sang.

[32] Hjelle B, Cyrus S, Swenson S, Mills R (1991). Serologic distinction between human T-lymphotropic virus (HTLV) type I and HTLV type II. Transfusion.

[33] Abrams A, Akahata Y, Jacobson S (2011). The prevalence and significance of HTLV-I/II seroindeterminate Western blot patterns. Viruses.

[34] Vandamme AM, Van Laethem K, Liu HF, Van Brussel M, Delaporte E, de Castro Costa CM (1997). Use of a generic polymerase chain reaction assay detecting human T-lymphotropic virus (HTLV) types I, II and divergent simian strains in the evaluation of individuals with indeterminate HTLV serology. J Med Virol.

[35] Santos TJT, Costa CMC, Goubau P, Vandamme AM, Desmyter J, Van Dooren S (2003). Western blot seroindeterminate individuals for Human T-lymphotropic Virus 1/2 (HTLV-I/2) in Fortaleza (Brazil): a serological and molecular diagnostic and epidemiological approach. Braz J Infect Dis.

[36] Césaire R, Bera O, Maier H, Lezin A, Martial J, Ouka M (2002). Seroindeterminate patterns and seroconversions to human T-lymphotropic virus type I positivity in blood donors from Martinique, French West Indies. Transfusion.

[37] Hjelle B, Mills R, Goldsmith C, Swenson S, Cyrus S (1992). Primary isolation of human T-cell leukemia-lymphoma virus types I and II: use for confirming infection in seroindeterminate blood donors. J Clin Microbiol.

